# Study on Reducing Water Absorption of Recycled Aggregates (RAs) by Microbial Mineralization

**DOI:** 10.3390/ma17071612

**Published:** 2024-04-01

**Authors:** Minglei Li, Haihe Yi, Yilin Su

**Affiliations:** 1 Department of Civil Engineering, School of Architecture and Engineering, Shandong University of Technology, Zibo 255000, China; 15842141818@163.com; 2 Department of Civil and Environmental Engineering, The Hong Kong Polytechnic University, Hung Hom, Kowloon, Hong Kong, China; seeyl.su@polyu.edu.hk

**Keywords:** recycled aggregates, microorganisms, mineralization deposition, aggregate water absorption, microstructure

## Abstract

Crushing waste concrete and using it directly as RAs has the disadvantages of high porosity and high water absorption. To achieve the reuse of resources, the researchers use microbial mineralization methods to further reinforce RAs. In this paper, the effect of the microbial carbonic anhydrase mineralization method on the water absorption of RAs was investigated, and the macroscopic analysis was performed by determining the indexes of water absorption and apparent density of RAs before and after the modification, and the microscopic analysis of RAs by using the methods of SEM, XRD, DSC, and EDS as well. According to the microscopic analysis, the mineralization products of microorganisms are calcium carbonate crystals, and with the increase in microbial liquid concentration, the water absorption rate of RAs shows a trend of decreasing and then increasing, and it can be found through the microscopic morphology that abundant mineralization products attached to the surface of the aggregate lead to the surface of the aggregate becoming rougher and more porous. The method of soaking the RAs in 3% bacterial solution and 0.1 mol/L calcium acetate solution followed by carbonation with 20% CO_2_ resulted in a 4.85% reduction in water absorption.

## 1. Introduction

With the acceleration of urbanization, construction activities have accelerated the decrease in natural aggregates and the increase in waste concrete production, resulting in a series of resource shortage and environmental problems. Recycling the waste is the best way to solve such problems, and has been a hot topic of research among scholars [1,2,3]. Using RAs from waste concrete to replace natural aggregates is an important means to realize sustainable development of resources and environment [4,5]. However, RAs have a high void ratio and water absorption [6,7], so the crushed RAs need to be further strengthened. Some researchers have enhanced the properties of RAs by acid treatment [8,9,10,11], mechanical grinding [12,13], and heat treatment [14]. However, these enhancement methods have the problems of environmental pollution and high energy consumption, which need to be further improved.

In addition, there are microbial mineralization and carbonation treatment reinforcement for RAs. Carbonation strengthening RAs process is mainly CO_2_ reacts with Ca(OH)_2_ and C-S-H gel in the regenerated aggregate mortar to produce CaCO_3_ and silica gel to fill the pores, thus achieving the purpose of strengthening the adherent mortar. The carbonation reaction formulae are shown in Equations (1) and (2) [15]. The pore structure, aggregate particle shape, and mechanical properties of RAs are improved after carbonation [16,17,18], and this strengthening process also plays a role in reducing carbon emissions [19]. Other properties, such as water absorption of RAs are also significantly reduce under CO_2_ carbonation, and the strengthening process of carbonation method is more energy-saving and environmentally friendly. However, the improved properties of RAs are still not as good as those of natural aggregates [20,21,22], and the carbonation strengthening process is slow.

Ca(OH)_2_ + CO_2_ → CaCO_3_ ↓ + H_2_O (1)

C-S-H + CO_2_ → SiO_2_·μH_2_O + CaCO_3_ ↓ (2)

**Figure 1:**
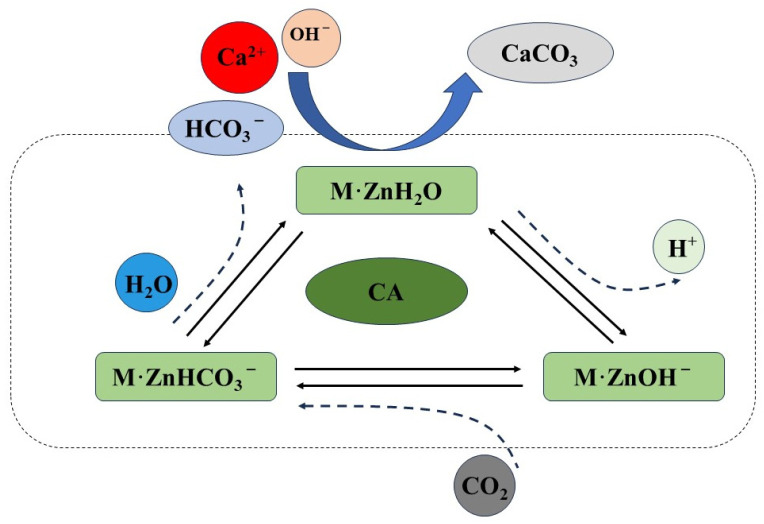
Schematic diagram of microbial mineralization mechanism.

Microbial mineralization can fill the pores and cracks on the surface of RAs effectively [23,24], and then reduce the water absorption of RAs [25]. Thus, microbial mineralization can obviously improve the properties of RAs and enhance the flexural tensile strength and compressive strength of RAs cast mortar [26,27]. Many scholars have improved the properties of RAs by microbial mineralization [28,29,30,31,32,33], and the microorganisms used are mainly urease bacteria. Urease bacteria can decompose urea into CO_2_ and NH_3_ under the action of its urease enzyme, and CO_2_ combines with Ca^2+^ under alkaline conditions to produce calcium carbonate crystals with the organism as the nucleation site. Although the modification effect is better, this process produces the harmful gas ammonia. Moreover, it has been found that certain non-urease bacteria can also accelerate the deposition of CaCO_3_, such as carbonic anhydrase microorganisms. Carbonic anhydrase can promote the reversible hydration reaction of CO_2_, which prompts the precipitation of Ca^2+^ as calcium carbonate crystals in an alkaline environment. The mineralization process of carbonic anhydrase is shown in Figure 1. In general, the microbial mineralization reaction of Ca^2+^ only by the RAs precipitation is not enough; one also needs to add an external calcium source to promote the reaction.

This study proposes a combination of microbial mineralization and accelerated carbonation to modify recycled aggregates. Carbonic anhydrase producing microorganisms were used to modify the RAs. The RAs were also maintained by different carbonation regimes. The macroscopic analysis of RAs was carried out through the indexes of water absorption and apparent density before and after the modification, and micro-properties of the modified RA were characterized by SEM, XRD, DSC and EDS. The effects of bacterial concentration on the water absorption of RAs were explored to determine the optimal modification method, and the mechanism of microbial mineralization on RAs were analyzed. After obtaining the optimal mineralization conditions, the concentration of mineralizable ions in the environment was optimized.

## 2. Materials and Methods

### 2.1. Materials

The RAs used in this study was made from laboratory-mixed C40 concrete with a size of 150 mm × 150 mm × 150 mm. The Portland cement used for the preparation of virgin concrete was produced by Zibo Shuangfeng Shanshui Cement Co. (Zibo, China). The mineral composition of the cement is shown in Table 1 and the C40 concrete mix ratio is shown in Table 2. After being crushed by a jaw crusher, the recycled concrete was divided into aggregates with different particle sizes according to the standard “Pebbles and crushed stones for construction” (GB/T14685) [34]. Based on the above method of crushing and screening the raw concrete, the concrete recycling rate for the preparation of usable RAs reached 85%. The sieving results are shown in Table 3. Recycled aggregates between 4.75 and 19.0 mm were taken for testing, water was taken from the laboratory tap water. The microorganism used was Bacillus sphaericus KW, and the bacterial powder was weighed proportionally to conFig the biological solution. After mixing well, the bacterial solution was placed in a thermostatic bed for incubation.

### 2.2. Methods

#### 2.2.1. Carbonation of Recycled Aggregate

**Figure 2:**
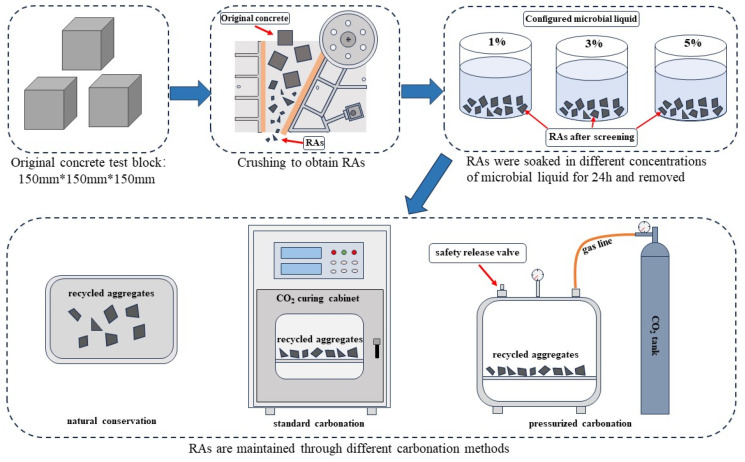
Process of RAs modification.

Figure 2 shows the process of recycled aggregate modification. In this test, the RAs were carbonated using three methods: natural conservation, standard carbonation in a carbonation cabinet, and pressurized carbonation in a closed vessel, respectively. The carbonation conditions are shown in Table 4.

#### 2.2.2. Microbial Mineralization

Firstly, the bacterial powder was weighed to prepare the microbial solution according to the proportion of 1%, 3%, and 5%, and put the prepared solution into the constant temperature shaker. Then, it was shaken and cultured under the rotational speed of 180 r/min and temperature of 30 °C for 30 h. At the end of the incubation, the bacterial solution in the conical flask was taken out, and the prepared regenerated aggregate was put into the bacterial solution to soak for 24 h and taken out.

#### 2.2.3. Optimization of Calcium Sources

After obtaining the optimal mineralization conditions, calcium ions in the environment were supplemented to analyze the effect of calcium sources on the performance of RAs. The RAs were immersed in 0.1 mol/L calcium acetate solution for 24 h and removed.

#### 2.2.4. Macro Performance Test

After the modification of RAs, the macroscopic properties such as water absorption and apparent density of RAs were determined according to Recycled Coarse Aggregate for Concrete (GB/T 25177-2010) [35].

#### 2.2.5. Optical Microscope Analysis

The surface morphology of the recycled aggregate was observed and analyzed by using an optical microscope. The surface morphology of recycled aggregates treated with different concentrations of bacterial solution and different carbonation methods were compared. The image is captured with a parameter resolution of 1920 × 1080, a magnification of 1000, and a frame rate of 30 FPS.

#### 2.2.6. Microanalysis

SEM and EDS: The prepared RAs were first soaked in anhydrous ethanol for 24 h and removed. The removed RAs were dried under vacuum at 65 °C to remove the free water in the samples, and the samples were plated with gold. The micro-morphological analysis of the microbiologically mineralized recycled aggregates was carried out by SEM, and the elemental composition of the mineralized products was analyzed by EDS.

XRD: The recycled aggregates were dried at 65 °C until constant weight. The dried RAs were pulverized to 80 μm for XRD analysis. The composition of the microbial mineralization products was qualitatively analyzed by a D8-02 polycrystalline X-ray diffractometer from Bruker-AXS, Germany. The parameters of the equipment were: 2θ range 5~90°, scanning speed 10(°)/min, step size 0.02°, and goniometric accuracy of 2θ ≤ ±0.01°.

DSC: Qualitative and quantitative analysis of the surface layer of recycled aggregate after microbial mineralization is carried out by the comprehensive thermal analyzer produced by TA Company in the United States. The parameters of the equipment: the temperature range is 25 °C~1400 °C, the heating rate is 0.1~50 K/min, the weighing range is 0~5 g, and the sensitivity of the balance is 0.1 μg.

BET: ASAP2020 specific surface area and porosity tester was used for the experiment. The sieved recycled aggregates between 4.75 and 19.0 mm were selected and put into the oven. It was dried at 105 °C for 2 h. The low-temperature liquid nitrogen adsorption experiment was carried out after cooling to room temperature. High purity liquid nitrogen (99.99%) was used for the experiment, the temperature was liquid nitrogen temperature, and the range of measured pore size was from 2 to 70 nm. The samples were degassed, the preset parameters were set, and the analytical process was carried out in a Dewar’s bottle.

## 3. Results and Discussion

### 3.1. Effect of Mineralization Conditions on Macroscopic Properties

**Figure 3:**
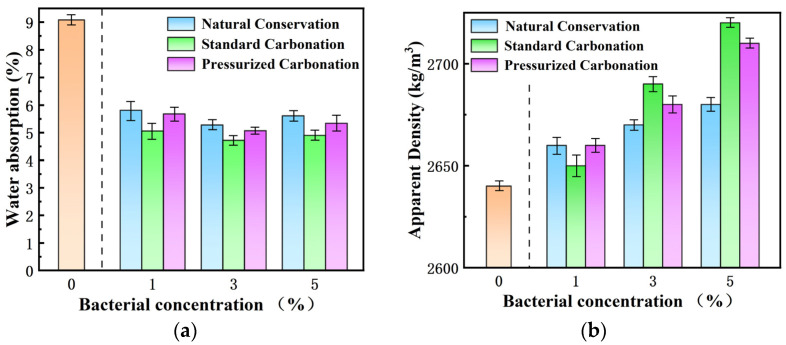
Comparison of water absorption (**a**) and apparent density (**b**) of modified RAs at different bacterial liquid concentrations.

The macroscopic properties of the RAs are shown in Table 5. The macroscopic properties of the RAs were all improved under microbial metabolism. The water absorption of modified S-1, S-2, S-3, P-1, P-2, and P-3 decreased to the range of 4.72% to 5.68%. As shown in Figure 3a, comparing the same conservation method, the water absorption of the RAs under the effect of three bacterial liquid concentrations of 1%, 3%, and 5% did not decrease with the increase in the bacterial liquid concentration, but showed a trend of decreasing and then increasing with the increase in the concentration of the bacterial liquid. The trend of increasing water absorption again may be because the mineralization of too high concentration of bacterial solution produces too much precipitate, and these oversized precipitates are attached to the surface of RAs thus making the surface rougher [36], together with the many nucleation sites produced by the microbial action leading to the generation of some micropores, so that there is a tendency for the water absorption of RAs to increase again. At the same time, comparing the water absorption rates of the three conservation methods under the same concentration of the bacterial solution, the water absorption rates of the bacterial solution-modified RAs were most significantly reduced by the accelerated carbonation of CO_2_ with a concentration of 20%. Moreover, after comparing the water absorption rate of three kinds of bacterial solution concentration under the same kind of conservation method, the bar graph can be more intuitively seen that the water absorption rate of the modified RAs shows a decreasing and then increasing trend with the increase in bacterial solution concentration, and the comparison found that the water absorption rate of the RAs modified by 3% bacterial solution is the lowest.

In terms of apparent density, as shown in Figure 3b. Under the effect of three microbial concentrations of 1%, 3%, and 5%, the apparent density of RAs treated with the same conservation method increased with the increase in bacterial concentration. In comparison with Figure 3a water absorption, after soaking in 1% concentration of bacterial solution, using 20% concentration of CO_2_ carbonation method has the lowest water absorption rate, and the corresponding apparent density is also lower than the other two carbonation methods. This is due to the faster precipitation of CaCO_3_ crystals on the pore surface under the standard carbonation method. This blocked CO_2_ transport to the interior of the pores [37] and inhibited the generation of CaCO_3_ crystals inside the pores, thus generating larger confined pores. The test group with the highest apparent density was M-6 with an apparent density of 2720 kg/m^3^, which is 2.26% higher than the apparent density of untreated recycled aggregate.

**Figure 4:**
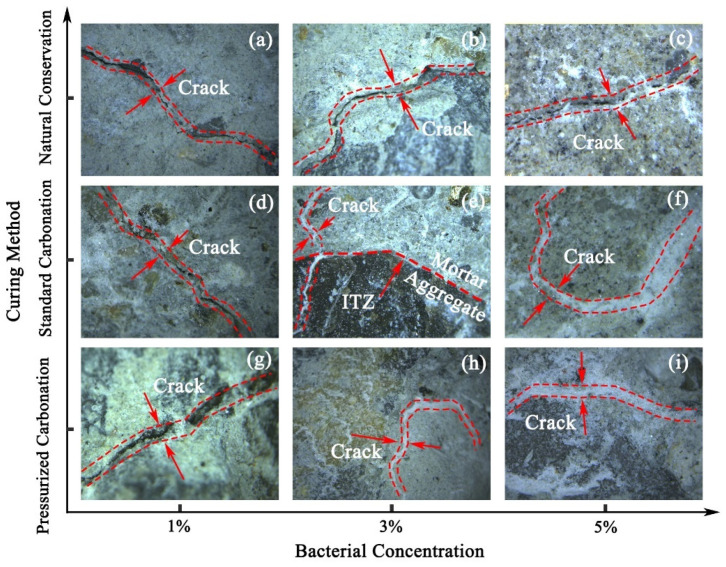
Surface defect morphology of modified RAs, (**a**) N-1, (**b**) N-2, (**c**) N-3, (**d**) S-1, (**e**) S-2, (**f**) S-3, (**g**) P-1, (**h**) P-2, (**i**) P-3.

The surface morphology of RAs under the optical microscope is shown in Figure 4. From the surface observation, it can be seen that the cracks on the surface of RAs were filled by white substances in varying proportions after modified by different concentrations of microorganisms. Along the direction of the horizontal axis of the bacterial liquid concentration gradually increased, the degree of white substance coverage is also greater and greater. When the concentration of the bacterial liquid was 1%, the filling effect of the gaps was not obvious, and only the generation of white substance could be observed; when the concentration of the bacterial liquid was 3%, the gaps were filled to different degrees, among which the filling effect was better after standard carbonization; when the concentration of the bacterial liquid was 5%, the gaps of the aggregates under the natural conditioning were filled. In the other two methods, the aggregate gaps were filled and more white material was spilled around the gaps. The overflow of white material can further prove the phenomenon that the water absorption rate decreases and then increases with the increase in the concentration of the bacterial solution.

Based on the analysis above, it could be concluded that the water absorption tended to be the lowest using the 20% CO_2_ carbonation conservation method at the same concentration of microbial modification. Therefore, this method was chosen for the conservation of RAs after modification in the later tests. According to the analysis of Figure 3, it was concluded that the RAs modified by 3% concentration of microorganisms had the lowest water absorption under the same carbonation method. Among them, the water absorption of S-2 was reduced to 4.72%, so the 3% concentration of the bacterial solution was selected for modification in the later test.

### 3.2. Effects of Calcium Ion Supplementation on Macroscopic Performance

**Figure 5:**
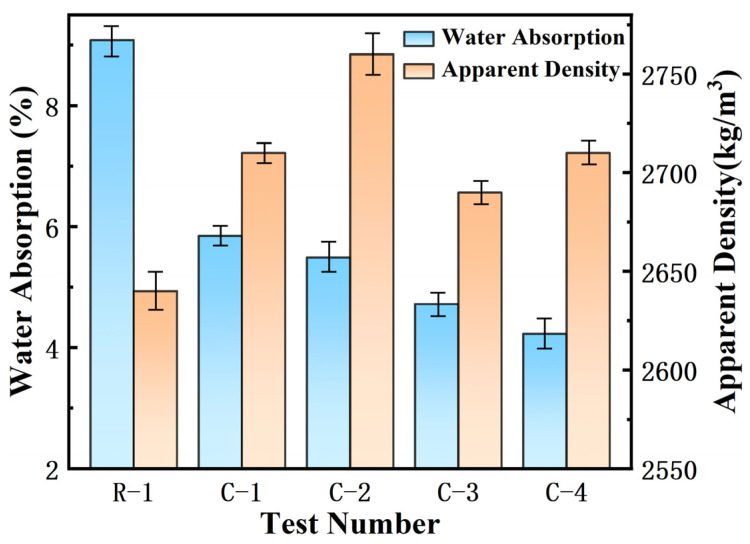
Comparison of water absorption and apparent density of modified RAs.

After determining the microbial concentration and conservation method, the calcium acetate solution was added as calcium supplements. The modified methods and corresponding macroscopic performances of RAs are shown in Table 6 and Figure 5, respectively. Compared to unmodified RAs, specimen C-1 showed a 35.6% decrease in water absorption, a 70 kg/m^3^ increase in apparent density, and a 2.26% increase in weight gain. Compared with RCA, specimen C-2 showed a 39.5% decrease in water absorption and a 4.55% increase in apparent density. The addition of microorganisms further reduced the water absorption of RAs, as shown in specimens C-3 and C-4. The change in apparent density is the same as analyzed above, the apparent density does not necessarily increase with decreasing water absorption, but rather there is some fluctuation.

**Figure 6:**
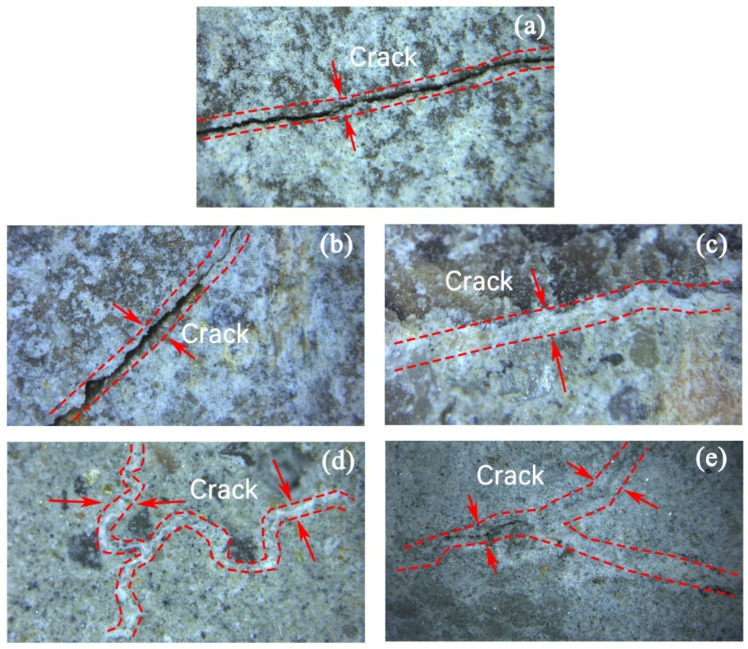
Surface morphology of RAs gap filling after modification, (**a**) R-1, (**b**) C-1, (**c**) C-2, (**d**) C-3, (**e**) C-4.

The fine morphology of gap filling of modified RAs is shown in Figure 6, where the gaps of each specimen were filled by the white substance. Generally, a better filling surface correspond to a lower water absorption. Among them, the C-4 test group showed the lowest water absorption, and the surface micro-cracks in Figure 6 were filled with white precipitates completely. The cracks are surrounded by white powdery solids and are accompanied by many transparent crystals.

### 3.3. Mineralization Products on the Surface of RAs

**Figure 7:**
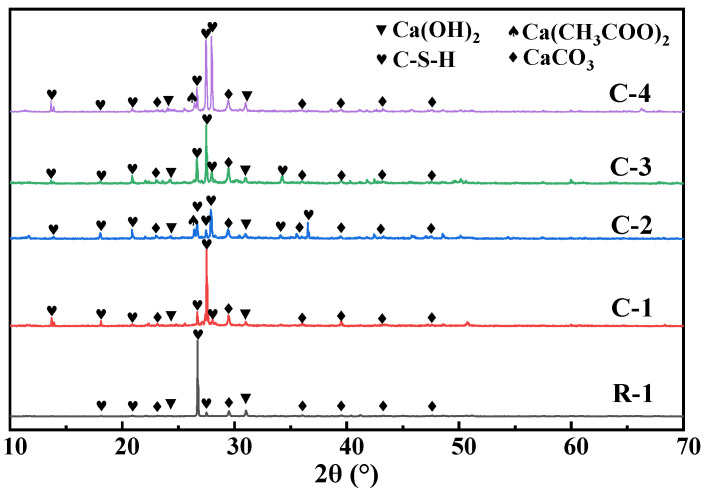
XRD patterns of surface products of RAs after treatment with different modifications.

Since the test material is an old mortar taken from the surface of recycled aggregate, there is an obvious hydrated calcium silicate characteristic peak present in the XRD pattern. From Figure 7, it is easy to see that after modification and carbonation by 3% concentration of the bacterial solution, a distinct calcite characteristic peak appeared at 2θ of 29.4°, which highly matched with the peak of calcite-type crystals in the standard PDF card. This suggests that after three concentrations of microbial treatment, the recycled aggregate surfacing mortar contained some amount of calcite crystals, in addition to the hydration products in the slurry. The effectiveness of the microbial mineralization method for modifying RAs was verified. Moreover, the substances on the surface of specimens C-3 and C-4 obtained after microbial mineralization were similar to R-1, which indicates that the composition is similar to that of RAs and MICP does not produce harmful substances in the recycled aggregates. Microorganisms accelerate the hydration reaction of CO_2_ through their production of carbonic anhydrase, which promotes the precipitation of Ca^2+^ as calcium carbonate crystals in an alkaline environment. The microorganisms provide nucleation sites on the surface of RAs for the precipitation of calcium carbonate crystals, causing the precipitated calcium carbonate crystals to aggregate on the surface of the aggregate.

**Figure 8:**
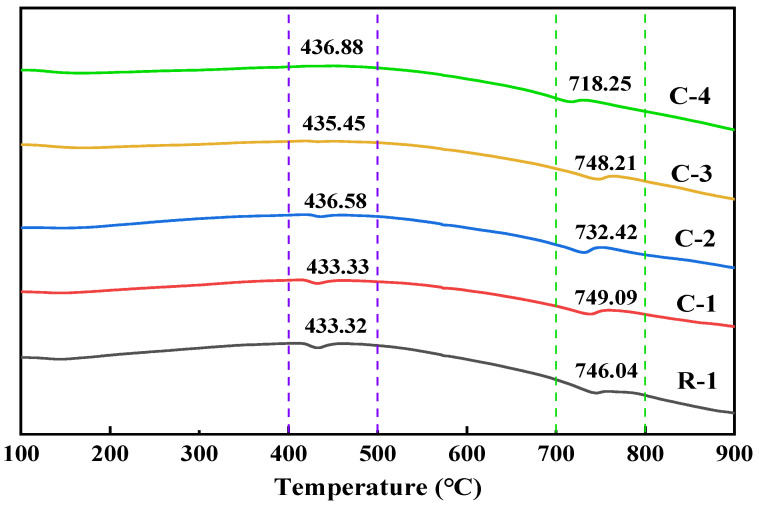
DSC profiles of surface products of RAs after treatment with different modification methods.

As can be seen from Figure 8, the DSC curves of specimens R-1, C-1, and C-2 showed obvious heat flow change peaks at 400 °C~500 °C and 700 °C~800 °C, which can be categorized as the heat-absorbing decomposition peaks of Ca(OH)_2_ and CaCO_3_. The changes in the DSC curves of the three specimens indicated that the RAs still contained a small amount of Ca(OH)_2_ in the old mortars C-1 and C-2 obtained after 20% CO_2_ carbonation treatment and soaking in calcium acetate solution. The Ca(OH)_2_ adsorption and decomposition peaks disappeared from the DSC curves of the old mortars on the surfaces of C-3 and C-4 modified by microbial soaking. This indicates that Ca(OH)_2_ in the recycled aggregate reacts with HCO_3_^−^ to form CaCO_3_ during microbial metabolism, resulting in a significant decrease in Ca(OH)_2_ content in the microbially modified old mortar of RAs. The DSC curves of specimens C-3 and C-4 showed obvious heat flow change peaks between 700 °C and 800 °C. It indicates that all the old mortars of RAs treated by microbial modification contain CaCO_3_. As the microbial mineralization reaction proceeds, the concentration of Ca^2+^ precipitated from the old mortar gradually decreases. Therefore, adding calcium acetate solution can replenish Ca^2+^ in the environment and promote the mineralization reaction.

**Figure 9:**
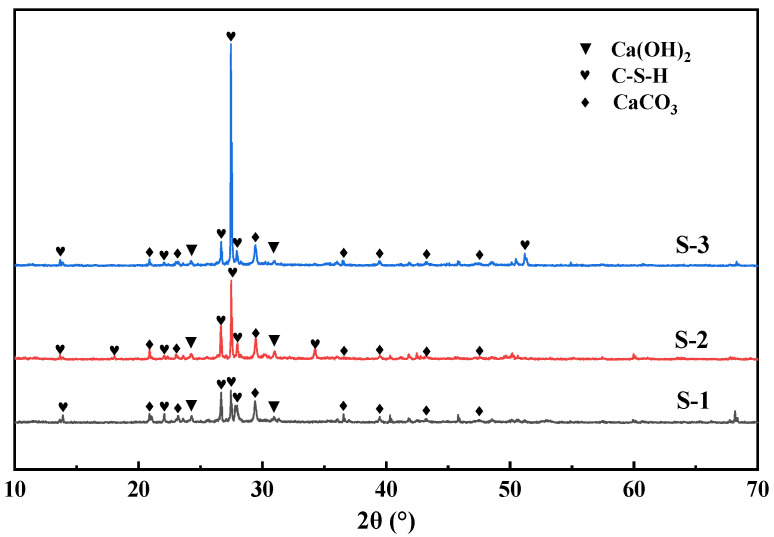
XRD patterns of surface products of RAs modified with different concentrations of bacterial solution.

**Figure 10:**
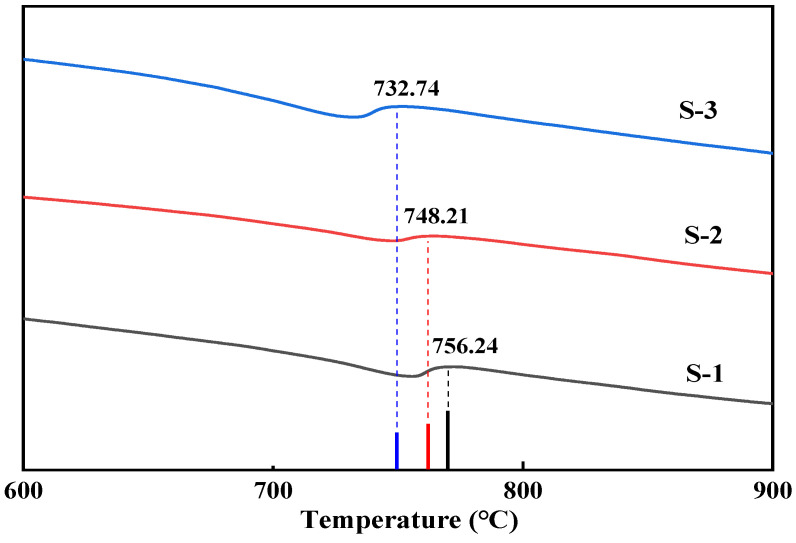
DSC analysis of surface products of RAs modified by different concentrations of bacterial liquid.

The mortar product compositions on the surface of RAs modified with different concentrations of bacterial solution under 20% CO_2_ carbonation and maintenance conditions are shown in Figure 9 and Figure 10. After the RAs were modified and carbonated with three concentrations of the bacterial solution, 1%, 3%, and 5%, there was an obvious calcite characteristic peak at 2θ of 29.4°. There is a high match with the peak of calcite-type crystals in the standard PDF card, and a certain amount of calcite crystals exist. From Figure 10, it can be seen that the surface products of RAs modified with different concentrations of bacterial solution under 20% CO_2_ carbonation conditions showed obvious CaCO_3_ heat-absorption decomposition peaks in the DSC curves at 700 °C~800 °C. This coincides with the results of the XRD analysis. It further indicates that CaCO_3_ crystals such as calcite are contained in the RAs surface mortar after treatment with the three concentrations of bacterial solution. It can also be seen from Figure 10 that the temperature of the heat-absorption decomposition of CaCO_3_ decreases slightly as the concentration of the bacterial solution increases. This may be a result of the RAs being under the same care and the increase in the concentration of the bacterial solution leading to an increase in the amount of CaCO_3_ attached to the surface of the aggregate. More exposed CaCO_3_ on the aggregate surface facilitates the decomposition when subjected to heat, so the higher the concentration of bacterial solution the lower the decomposition temperature.

**Figure 11:**
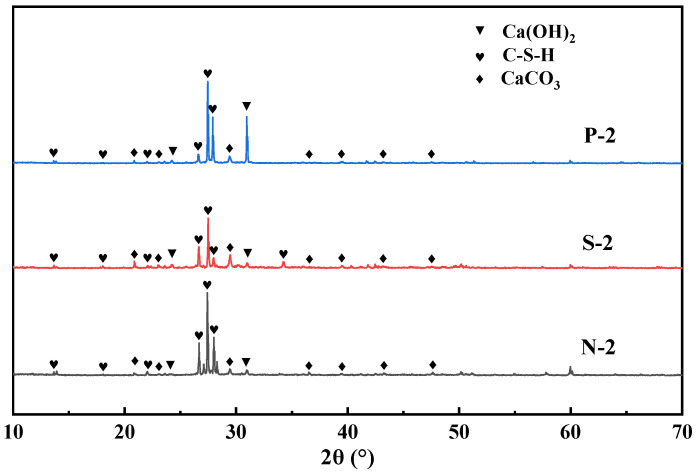
XRD patterns of surface products of RAs under different maintenance regimes.

**Figure 12:**
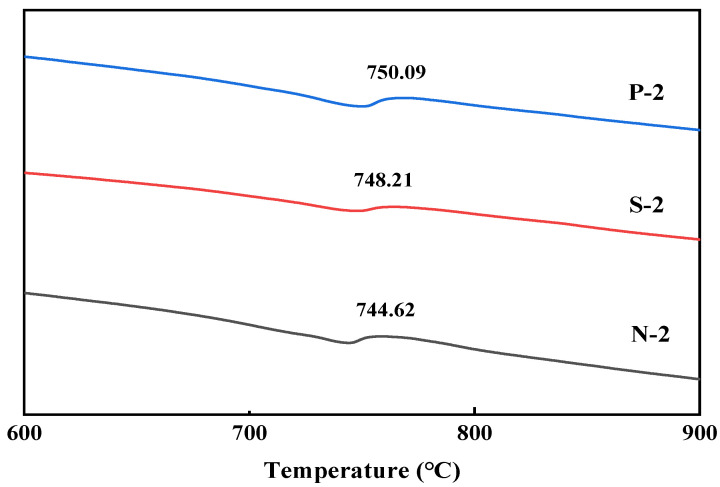
DSC analysis of surface products of RAs under different maintenance methods.

As can be seen from Figure 11, there are obvious characteristic peaks of hydrated calcium silicate after treatment by different modification methods. There is an obvious characteristic peak at 2θ of 29.4°, which matches well with the peak of the standard PDF card of calcite-type crystals. It indicates that a certain amount of calcite crystals existed in the microbially modified recycled aggregate surface mortar under three different maintenance regimes. As can be seen in Figure 12, after microbial mineralization of the RAs, there is an obvious peak of heat-absorption decomposition of calcium carbonate at 700 °C~800 °C. This is consistent with the results of XRD analysis, which further indicates that the surface mortar compositions of microbial-modified RAs under different maintenance regimes are similar and all contain calcite crystals.

XRD and DSC analyses showed that the white substance produced by mineralization was CaCO_3_ precipitation, so the addition of 0.1 mol/L calcium acetate as a calcium source could effectively promote the carbonation reaction. It can be well reflected by the comparison graph of water absorption of specimens. The substances produced under different maintenance conditions and different bacterial liquid concentrations of RAs were analyzed, and the products were found to be the same. It can be shown that the products generated by microbial mineralization are not affected by the concentration of bacterial liquid and the maintenance method.

**Figure 13:**
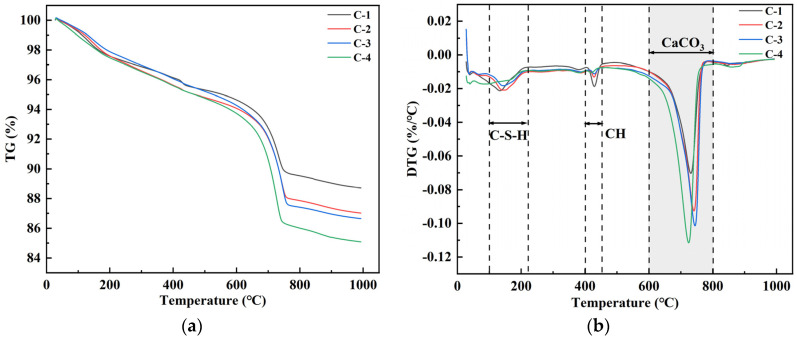
TG and DTG curves of the recycled aggregates under different modification conditions: (**a**) TG curves; (**b**) DTG curves.

Figure 13 shows the TG and DTG curves of the recycled aggregates under different modification conditions. The weight loss in the range of 100 °C~200 °C corresponds to the C-S-H gel dehydration; the weight loss in the range of 400 °C~450 °C corresponds to the Ca(OH)_2_ dehydration; and the weight loss in the range of 600 °C~800 °C corresponds to the decomposition of CaCO_3_, which further verifies the above analysis of the surface productions of the recycled aggregates. The CaCO_3_ content can be calculated from the weight loss rate. The CaCO_3_ contents of C-1, C-2, C-3, and C-4 were 11.6%, 14.1%, 15.6, and 17.5%, respectively. There was a significant increase in calcium carbonate production with the addition of calcium sources and microorganisms under standard carbon dioxide conservation. However, the amount of calcium carbonate in C-3 was greater than that in C-2 under the same condition of conservation. This was due to the catalytic effect of enzymes in microorganisms and the attraction of Ca^2+^ by the negative charge carried by the microorganisms, which led to a more efficient nucleation of CaCO_3_. This indicates that the addition of microorganisms affects CaCO_3_ production to a greater extent than the addition of calcium sources. With the simultaneous addition of microorganisms and calcium source, the CaCO_3_ content was increased by 6.9% compared to standard maintenance only, which has a better potential for carbon sequestration.

### 3.4. Micro-Structure of RAs

**Figure 14:**
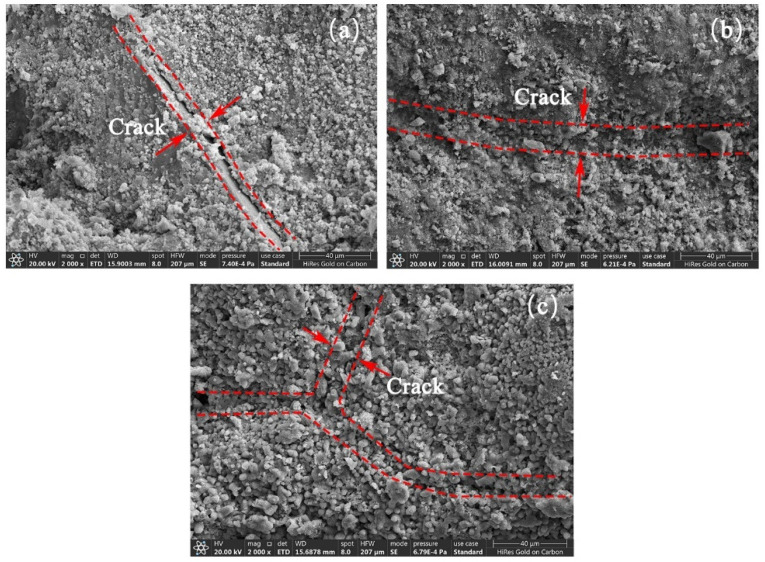
Surface microscopic morphology of RAs modified by different concentrations of bacterial liquid (magnified 2000 times), (**a**) S-1, (**b**) S-2, (**c**) S-3.

From Figure 14, it is easy to see that the bacterial solutions at 1%, 3% and 5% concentrations have different modification effects on the surface of recycled aggregates. CaCO_3_ crystals were evidently generated near the cracks on the surface of Specimen S-1, but filled the gap to a lesser extent. Specimen S-2 The surface cracks were covered and a few cracks were not filled. The vicinity of the cracks is better covered by CaCO_3_ crystals and the aggregate surface is more dense. Specimen S-3 surface cracks were mostly filled with CaCO_3_ crystals, and a large amount of mineralization products were generated inside the cracks and in the nearby area. As the concentration of bacterial solution increased, CaCO_3_ crystals gradually repaired the cracks on the surface of RAs and gradually overflowed at the cracks. Under excessive concentration of microbial mineralization, the overflowed CaCO_3_ crystals as well as the nucleation sites formed by microorganisms led to a rougher surface of RAs. Therefore, it further explains that the water absorption of RAs produces a tendency of decreasing and then increasing.

**Figure 15:**
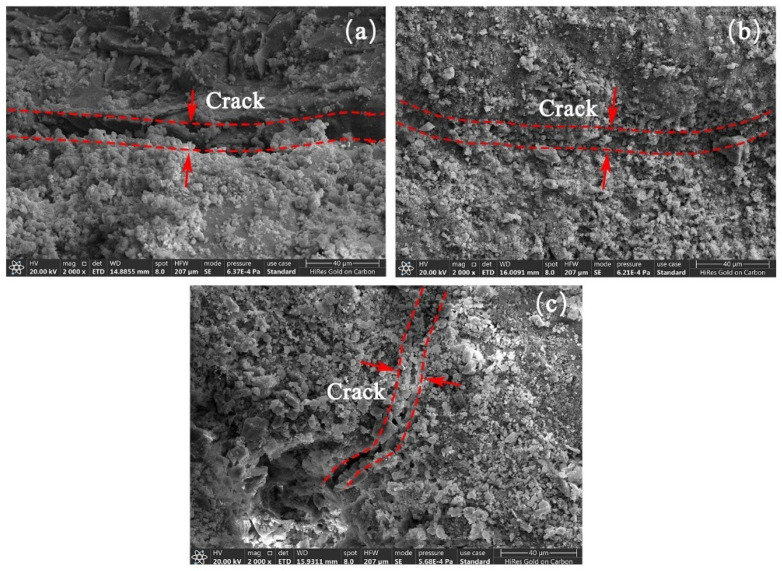
Surface microscopic morphology of modified RAs with different conservation methods (magnified 2000 times), (**a**) N-2, (**b**) S-2, (**c**) P-2.

From Figure 15, it is easy to see that the surface cracks of specimen N-2 and specimen P-2 are well-filled by microbial mineralization products. However, a few cracks still exist. The surface cracks of specimen S-2 were filled and had a dense structure.

**Figure 16:**
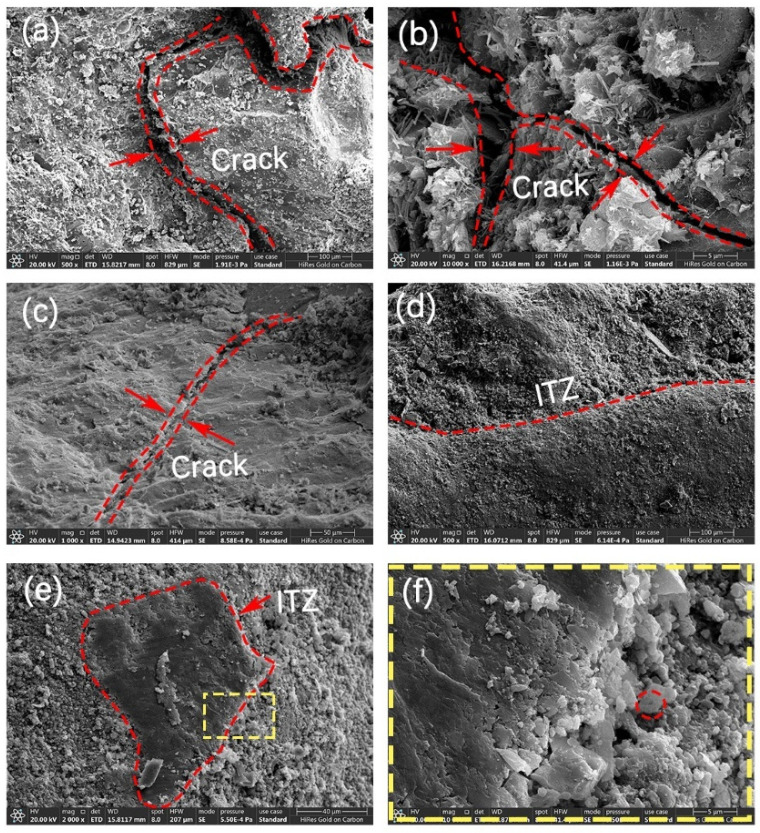
Micro-morphology of RAs, (**a**) R-1, (**b**) C-1, (**c**) C-2, (**d**) C-3, (**e**) C-4; (**f**) is a magnified view of C-4.

Comparison of the microscopic morphology of four kinds of recycled aggregates, R-1, C-1, C-2, and C-3, is shown in Figure 16. Observation of specimen R-1 by SEM reveals that there are obvious cracks on the surface of the aggregate with a loose structure, as shown in Figure 16a; as shown in Figure 16b, needle-like CaCO_3_ crystals are generated near the cracks of specimen C-1. The crystals mainly show rhombic and granular structures. However, the degree of filling the cracks is small; as in Figure 16c, the cracks of specimen C-2 are filled by CaCO_3_ crystals with a more dense structure; Figure 16d shows the ITZs after microbial immersion at a concentration of 3% + standard carbonation. Nucleation sites formed by microorganisms can be observed on the surface of the cracks and in the vicinity. The cracks were covered and filled by CaCO_3_ crystals generated by microbial mineralization; Figure 16e shows C-4 after 0.1 mol/L calcium acetate solution + 3% concentration microbial immersion + standard carbonation. The light-colored area is old mortar, and the dark-colored and flat part is aggregate. Nucleation sites of microorganisms appeared near the cracks. The cracks are filled with CaCO_3_ crystals and have a dense structure, which reduces the water absorption of the RAs.

**Figure 17:**
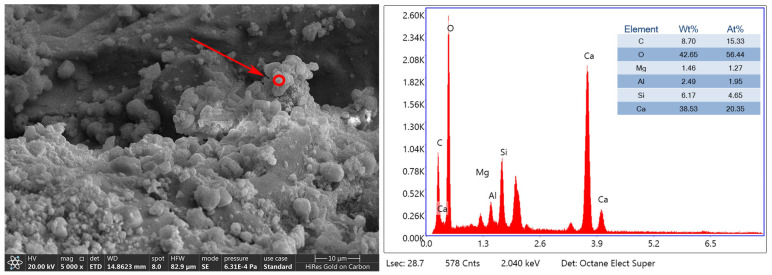
Energy spectrum analysis of microbial mineralization products on the surface of C-3: The red circle indicated by the arrow is the EDS scan position.

Figure 17 shows the micro-morphology of the surface of C-3 aggregate and the energy spectrum analysis of the surface generators, from which it can be seen that a large number of irregularly shaped crystals existed on the surface of RAs first soaked by 3% concentration of microorganisms, and then carbonated by 20% CO_2_ stacked growth with a dense structure. The energy spectrum analysis shows that the ratio of Ca, C, and O is close to 1.3:1:3.7; the element Si accounts for 4.65%; the ratio of Ca, O, and C in CaCO_3_ is 1:3:1; and the crystals inside the cracks and on the surface of the aggregates are more densely grown. The crystals are interspersed with each other, and the cracks are filled with calcium carbonate crystals.

**Figure 18:**
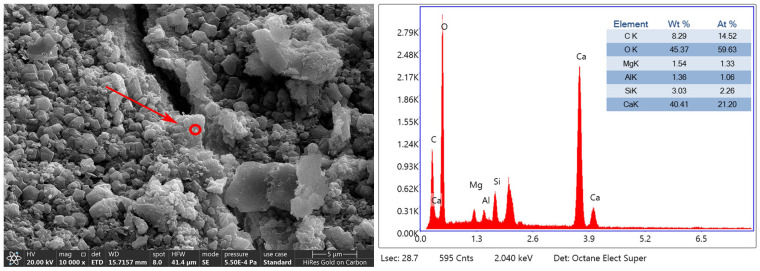
Energy spectrum analysis of microbial mineralization products on the surface of C-4: The red circle indicated by the arrow is the EDS scan position.

Figure 18 shows the micro-morphology of the aggregate surface and the energy spectrum analysis of the surface generators of group C-4. The RAs were treated with a 3% concentration of microorganisms soaking the RAs while adding 0.1 mol/L calcium acetate, and then 20% CO_2_ conservation. A large number of irregular crystals appeared on the surface of the RAs to accumulate and grow and cover the surface of the aggregates. The aggregate surface had a dense structure with the production of massive calcite generated by microbial mineralization. The energy spectrum analysis shows that the ratio of Ca, C, and O is close to 1.5:1:4.1, the element Si accounts for 2.26%, and the ratio of Ca, O, and C in CaCO_3_ is 1:3:1. The growth of crystals inside the cracks and on the surface of the aggregate is relatively dense. The crystals are interspersed with each other, the growth is dense, and some microcracks are filled with calcium carbonate crystals.

**Figure 19:**
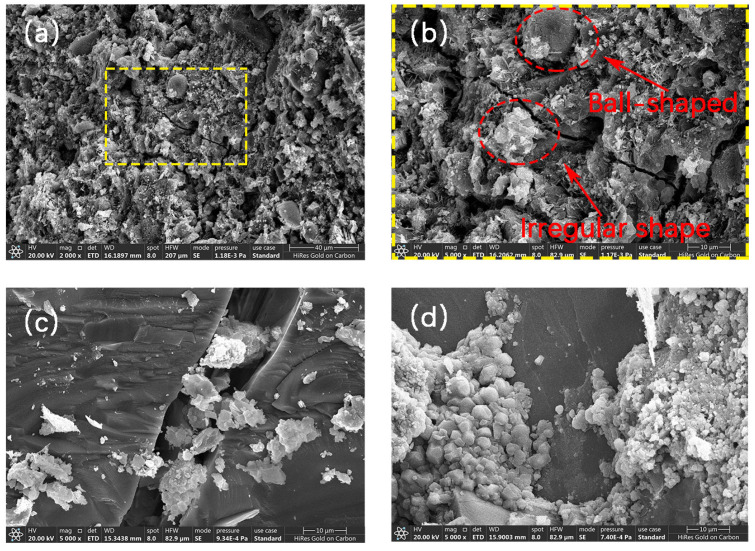
Particle morphology of mineralization products: (**a**) mineralization products under microbial action, (**b**) local magnification of (**a**), (**c**) particle aggregation phenomenon under standard carbonation, and (**d**) particle aggregation phenomenon under microbial action.

In this study, the shape and changes in calcium carbonate crystals on the particle scale were observed by scanning electron microscopy. According to Figure 19a,b, it can be seen that the shape of calcium carbonate particles generated by microorganisms is spherical and irregular, and the particle size is about 5–10 μm and, according to the literature [38], the particle size of calcium carbonate will also increase gradually with the extension of mineralization time. From Figure 19b, it can also be found that the surface of calcium carbonate particles is relatively rough, which is due to the participation of microorganisms in the nucleation process of crystals. Products such as polysaccharides on the surface of the bacteria are in the form of gels, which leads to a rough surface of the particles. From Figure 19c,d, it can be seen that calcium carbonate particles of different shapes will aggregate to form clusters. And the aggregation after microbial action will be more obvious than that after standard carbonation. This may be due to the fact that under the microbial charge, calcium ions are attracted and the aggregation of calcium carbonate occurs more easily.

### 3.5. Pore Structure of RAs

It can be observed by SEM that the large pores on the surface of RAs are effectively filled by CaCO_3_ crystals under the action of microbial mineralization. However, there are still many micropores in the aggregate, which are not easily observed under SEM analysis. And, since micropores in aggregates have an effect on water absorption and are often overlooked, nanopores were tested in this experiment. The MIP method is more advantageous for large pore testing, but the determination of nanoscale pores is not comprehensive and accurate, and the porosity determined by the MIP method is often lower than the porosity of the actual aggregate [39]. In contrast, the nitrogen adsorption method is effective for the determination of pore sizes smaller than 100 nm [40].

**Figure 20:**
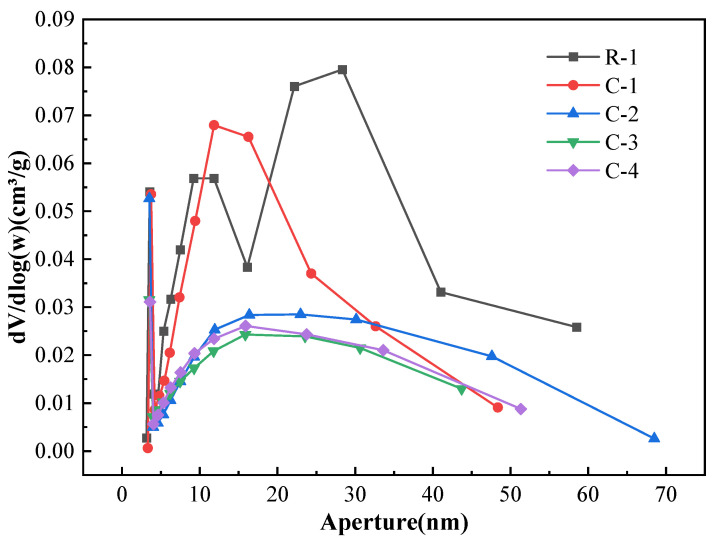
Pore distribution of modified RAs.

The RAs pore distribution was analyzed by the method of BET, as shown in Figure 20. The R-1 pores of the specimen were mainly distributed between 5~40 nm pore size, and the pore volume was the largest when the pore size was between 20~30 nm; the pore structure curve of the regenerated aggregate was shifted to the left after carbonation. The pore volume of the regenerated aggregate was the largest in the interval of 10~25 nm. It indicates that the pore size of regenerated aggregate is reduced after carbonation, and the pores are mainly distributed in the range of 10~25 nm; after RAs are soaked through 0.1 mol/L calcium acetate and carbonated by 20% CO_2_. The pore curve was smooth and the pore size distribution was uniform. It shows that 0.1 mol/L calcium acetate can effectively improve the pore structure of recycled aggregate. CaCO_3_ crystals fill the large pores of aggregate and make the pore distribution more uniform, so the water absorption rate is greatly improved; the pore structure curves of RAs treated by C-3 and C-4 modification methods in Figure 20 are similar, and are smoother than that of A-2 group. The two curves of C-3 and C-4 are shifted to the lower left overall between 5~70 nm, indicating that the pore structure of recycled aggregate is further improved after microbial modification. The pore diameter was further reduced, the pore distribution was more uniform, and the water absorption was further improved.

### 3.6. Modification Mechanism of Carbonic Anhydrase Producing Microorganisms

**Figure 21:**
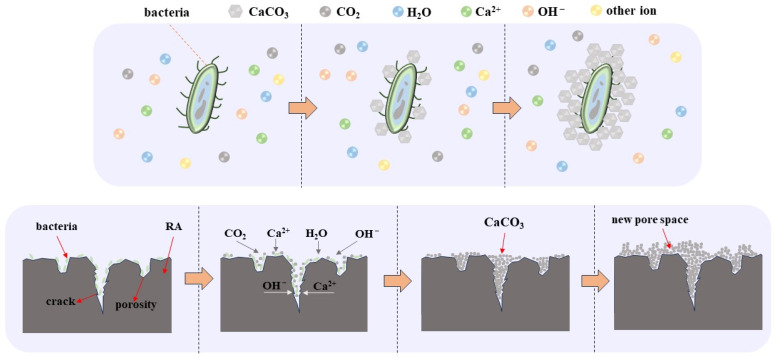
Mechanism of microbial mineralization to improve RAs water absorption.

The mechanism of microbial mineralization to improve the water absorption of RAs is shown in Figure 21. When the microbial solution soaks the RAs, the microorganisms attach to the surface of the aggregate as well as to the pores. The microorganisms accelerate the hydration reaction of CO_2_ by producing enzymes, then promote the reaction between calcium ions and carbonates The generated CaCO_3_ crystals are adsorbed on the microbial surface, thus filling the surface gaps and improving the water absorption of RAs. However, excessive CaCO_3_ crystal generation leads to the creation of new porous structure on the RA surface, which lead to an increase in water absorption.

### 3.7. Economic Analysis

Recycling waste concrete and using it as a substitute for natural aggregate can significantly reduce problems such as construction waste storage. At the same time, RAs can also play the role of carbon sequestration in the process of modification, which promotes the sustainable development of resources and the environment. In this paper, microbial mineralized aggregate modification has been investigated, on the basis of which an economic analysis of the production of RA has been carried out.

The cost comparison between natural aggregate and recycled aggregate is shown in Table 7. The market price of natural sand and gravel is about 106 RMB/ton, while the recycled aggregate processor needs to charge about 20 RMB from the builder for each ton of construction waste processed. Natural aggregates can be purchased and put into use directly without any other costs. The production of recycled aggregates involves crushing, screening, transportation, and modification costs. The combined cost of equipment and electricity is about 10.4 RMB/ton. The cost of road transportation is 30~40 RMB/ton (70 km), and the combined cost of transportation and labor is 100 RMB/ton. The CO_2_ concentration in the exhaust gases of some factories is between 18% and 23%, which can provide carbonization conditions for RAs conservation. For the production of RAs, the cost of transportation and labor is the main bottleneck, and how to choose the right disposal site is key. In conclusion, the cost difference between natural and recycled aggregates is not significant and has good economic benefits. At the same time, the production and use of RAs can greatly alleviate the impact of solid waste on the environment and play a certain role in carbon sequestration.

## 4. Conclusions

(1) The water absorption of RAs modified with 3% microbial concentration and then carbonated by 20% CO_2_ was minimized. The water absorption of RAs showed a fluctuating trend, first decreasing and then increasing with bacterial concentration. According to the micro-structure analysis based on optical microscopy and SEM, excess bacteria may produce excess crystals on the aggregate surface, which then form more capillaries due to heterophase nucleation of the bacteria. Too much nucleation sites produced by microbial mineralization can lead to a rougher and more porous RA surface, which again increases the water absorption of the aggregate.
(2) According to the mineralization mechanism of microorganisms, supplementation of calcium ions can promote the mineralization reaction. RAs mineralized by microorganisms at 3% concentration reduced water absorption to 4.72% and increased apparent density by 1.89%. The addition of 0.1 mol/L calcium acetate decreased the water absorption to 4.23% and increased the apparent density by 2.65%. After supplementing the calcium source, the water absorption decreased to 10.4% and the apparent density increased by 0.74%.
(3) The mineralization products on the RA surface were found to be identical by XRD and DSC analyses, implying that the microbial-assisted carbonation modification was stable. The analysis concluded that the microbial mineralization products were mainly CaCO_3_ crystals, which did not adversely affect the recycled aggregate surface mortar.
(4) SEM and BET analyses yielded that the cracks on the surface of RAs were effectively repaired after microbial mineralization. The larger pores on the aggregate surface were effectively filled by CaCO_3_ as observed by SEM. From the distribution analysis of nanopores, the large micropores or gap were filled and the micropores was further improved. The whole surface structure of RAs tends to be smooth after microbial mineralization.

## Figures and Tables

**Table 1:** Mineral composition of cement.

Components	C_3_S	C_2_S	C_3_A	C_4_AF	f-CaO	Other
Content (%)	54.8	22.86	8.03	9.53	0.91	3.87

**Table 2:** C40 concrete mixing ratio.

Water-Binder Ratio	Sand Ratio	Water Consumption (kg/m^3^)	Cement (kg/m^3^)	Sand (kg/m^3^)	Stone (kg/m^3^)
0.49	0.465	195	399	623	1183

**Table 3:** Screening results of RAs.

Particle Size	26.5	19.0	16.0	9.5	4.75	≤4.75
Content (%)	5.8	18.7	11.3	30.5	19.0	14.5

**Table 4:** Parameters of carbonation conditions.

Carbonation Method	Pressure (KPa)	Humidity (%)	Temperature (℃)	CO_2_ Concentration (%)	Carbonation Duration (h)
Natural Conservation	101	70 ± 5	25 ± 5	0.03	5
Standard Carbonation	101	70 ± 5	25 ± 5	20	5
Pressurized carbonation	300	70 ± 5	25 ± 5	99.9	5

**Table 5:** Summary of modification methods for RA specimens (1).

Test Number	Modification Methods	Conservation Methods
R-1	—	—
N-1	1% bacterial liquid	Natural Conservation
N-2	3% bacterial liquid	
N-3	5% bacterial liquid	
S-1	1% bacterial liquid	Standard Carbonation
S-2	3% bacterial liquid	
S-3	5% bacterial liquid	
P-1	1% bacterial liquid	Pressurized Carbonation
P-2	3% bacterial liquid	
P-3	5% bacterial liquid	

**Table 6:** Summary of modification methods for RA specimens (2).

Test Number	Modification Methods	Carbonation Methods
R-1	—	—
C-1	—	Standard Carbonation
C-2	0.1 mol/L calcium acetate	Standard Carbonation
C-3	3% bacterial liquid	Standard Carbonation
C-4	3% bacterial liquid + 0.1 mol/L calcium acetate	Standard Carbonation

**Table 7:** Costs of Natural Aggregates (NAs) and Recycled Aggregates (RAs).

Aggregate Type	Aggregate Unit Price (RMB/ton)	Electricity Cost of Equipment (RMB/ton)	Bacteria Cost (RMB/ton)	Cost of Transportation and Labor (RMB/ton)	Total (RMB/ton)
NAs	106	—	—	—	106
RAs	−20	10.4	25	100	115.4

## Data Availability

Data are contained within the article.
